# Stem Cell Therapy and Innate Lymphoid Cells

**DOI:** 10.1155/2022/3530520

**Published:** 2022-08-02

**Authors:** Divya Verma, Mukesh Verma, Rangnath Mishra

**Affiliations:** ^1^Division of Allergy & Immunology, Department of Medicine, National Jewish Health, Denver, USA; ^2^Global Institute of Stem Cell Research and Therapy, San Diego, USA

## Abstract

Innate lymphoid cells have the capability to communicate with other immune cell types to coordinate the immune system functioning during homeostasis and inflammation. However, these cells behave differently at the functional level, unlike T cells, these cells do not need antigen receptors for activation because they are activated by the interaction of their receptor ligation. In hematopoietic stem cell transplantation (HSCT), T cells and NK cells have been extensively studied but very few studies are available on ILCs. In this review, an attempt has been made to provide current information related to NK and ILCs cell-based stem cell therapies and role of the stem cells in the regulation of ILCs as well. Also, the latest information on the differentiation of NK cells and ILCs from CD34+ hematopoietic stem cells is covered in the article.

## 1. Introduction

The innate immune responses are the first line of defense against invading pathogens and then initiate specific adaptive immune responses. These responses rely on the body's ability to recognize conserved features of pathogens even though those are not previously exposed to the host. This preliminary evolutionary defense strategy, relatively speaking, is the dominant immune response found even in plants, fungi, insects, and primitive multicellular organisms [1]. The major functions of the vertebrate innate immune system are for recruiting immune cells to the sites of infection through the production of chemical factors including specialized chemical mediators allied cytokines, activating the complement cascade to identify bacteria, activating cells to promote clearance of antibody complexes or dead cells, and identify and remove foreign substances present in organs, tissues, blood, and lymph, by specialized white blood cells. Functions like activation of the adaptive immune system through antigen presentation and acting as a physical and chemical barrier to infectious agents are also carried out by this system [2].

Innate lymphoid cells (ILCs), the major functional active component of the innate immune system, play important roles in fending off infections, and thereby, in controlling inflammation and diseases [2, 3]. ILCs, which lack Rag- (recombination-activating gene-) mediated antigen receptors' recombination ability [4, 5], are globally distributed throughout the body but more in the mucosal surface tissues [6]. These cells have the capability to communicate with other cell types of the body to coordinate the immune system functioning during homeostasis and during inflammation [4, 5, 7].

**Figure 1:**
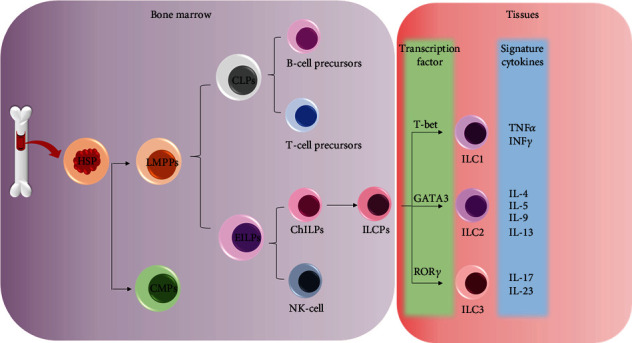
Differentiation of ILCs from hematopoietic stem cells (HSCs): HSCs differentiate into lymphoid-primed multipotent progenitors (LMPPs) and common myeloid progenitors (CMPs). LMPPs further differentiate into early innate lymphoid progenitors (EILP) and common lymphoid progenitors (CLPs). CLPs give rise to T and B cells whereas ELPs differentiate into common helper ILC progenitors (CHILPs) and NK cells. CHILPs further differentiate to produce PLZF-dependent ILC1s, ILC2s, and ILC3s.

ILCs develop from the hematopoietic stem cells (HSCs) in several regulated steps (Figure 1). HSCs first differentiate into hematopoietic multipotent progenitors including common myeloid progenitors and lymphoid primed multipotent progenitors (LMPPs) [8, 9]. Next, LMPPs differentiate into common lymphoid progenitors (CLPs) and early innate lymphoid progenitors (EILPs). CLPs give rise to B cell and T cell precursors while EILPs give rise to natural killer cell precursors (NKPs) and to common helper ILC precursors (ChILPs) [10, 11]. CHILPs give rise to promyelocytic leukemia zinc finger (PLZF) expressing ILCPs [12, 13] which give rise to all ILC groups except NK cells or lymphoid tissue inducer (LTi) cells. ILCs can be recognized by their classical lymphoid cell-like morphology and by other biochemical characters which include none of the major surface molecules, designated as cell lineage marker negative (Lin^−^); it can vary from species to species. In humans, the cell surface markers are CD3, CD19, CD56, CD68, CD205, and FceR1 while in mice, these are CD3, Ly-6G/Ly-6C, CD11b, CD45R/B220, TER-119/erythroid cells, NK1.1, and FceR1a. These cell surface molecules also help to recognize other immune cell types [5, 14, 15]. Based on the expressed cytokines and transcription factors, these cells are divided into three different ILC subsets [16, 17]. ILC1s are characterized by the expression of transcription factor T-bet and by the secretion of cytokines Th1 and IFN-*γ*. ILC2s, which express the transcription factor GATA-3 and produce Th2-type cytokines, respond to enormous extracellular pathogens. ILC3s, which express transcription factor RoRyt and produce Th17- and Th22-like cytokines, are required for host defense against extracellular bacteria and fungi. Though all ILCs are derived from HSCs but little is known about the role of ILCs in stem cell therapy and regulation of ILCs by stem cells during diseases.

## 2. NK Cells and Stem Cells

**Figure 2:**
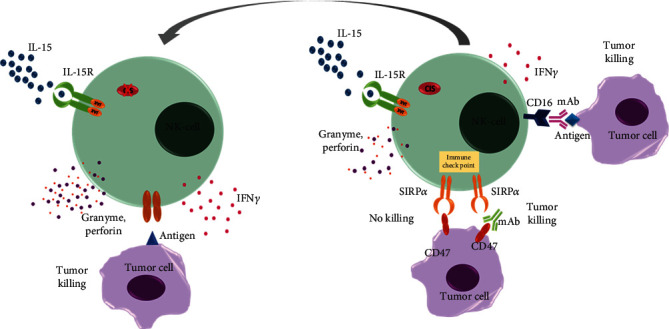
NK cell-mediated cell toxicity: NK cells can be derived from different sources like PB, UCB, ESCs, and CD34+ HSCs, and also from induced pluripotent stem cells (iPSCs). In the presence of CIS, NK cell cytotoxicity is blocked by inhibition of IL-15 and JAK/STAT signaling pathways. In the absence of CIS, NK cells produce more granzyme B and IFN-*γ* which are involved in tumor eradication. Antibody-mediated blocking of CD47 and CD16 is important for tumor killing; otherwise, they interact with their receptors and send “do not eat me” messages to NK cells and tumors continue to grow.

Natural killer (NK) cells, also known as large granulocytes, are specialized immune effector cells that play a critical role in immune activation against abnormal cells are also important for the immune surveillance. NK cells, which can be used for NK cell-based immunotherapy in cases of cancer, can be differentiated from stem cells from several sources like peripheral blood cells (PBCs), umbilical cord blood (UCB), embryonic stem cells (ESCs), CD34+ hematopoietic stem cells (HSCs), and induced pluripotent stem cells (iPSCs) [18]. However, the cells of different origins have their own limitations. For example, the abilities of the NK cells generated using allogeneic peripheral blood (PB) are donor-dependent and have heterogeneous activities indicating that the activities differ from donor to donor. UCB-derived NK cells also have donor-specific activities and are needed to be expanded before use. Recently, Goldenson et al. have reported that UMB and iPSC-derived natural killer cells have differences in their cytotoxic activity and KIR profiles [19], which is a very important information for the selection of the most effective NK cell populations for therapeutic purposes. iPSCs are also efficiently differentiated to produce mature NK cells (iPSC-NK cells) [20, 21], and these can be easily engineered by existing genome editing tools. Thus, iPSCs provide an important platform to produce NK cells with an improved population of homogenous cells having anti-tumor activity, a property extremely desired for treating solid tumors [21–23]. Studies have identified novel NK cell-specific chimeric antigen receptors (CARs) that mediate improved killing of ovarian cancer cells both in vitro and in vivo [21]. iPSCs used to create an unending source of induced pluripotent stem cell-derived NK (hnCD16-iNK) cells of human origins which have improved antibody-dependent cell-mediated cytotoxicity (ADCC) and are efficient in the killing of cells of solid tumors as well as those causing hematologic malignancies (Figure 2) [23]. CD16, a molecule of the immunoglobulin superfamily (IgSF) involved in antibody-dependent cellular cytotoxicity (ADCC), is found on the surface of natural killer cells. However, recently, some researchers generated a triple-gene-edited induced pluripotent stem cell (iPSC) [24]. They engineered a clonal iPSC line to express a high-affinity, noncleavable version of the Fc receptor CD16a and a membrane-bound interleukin- (IL-) 15/IL-15R fusion protein. Their third edit was a knockout of the ectoenzyme CD38, which hydrolyzes NAD+. Natural killer (NK) cells derived from these uniformly engineered iPSCs, termed iADAPT, displayed metabolic features and gene expression profiles mirroring those of cytomegalovirus-induced adaptive NK cells. iADAPT NK cells persisted in vivo in the absence of exogenous cytokine and elicited superior antitumor activity [24]. These findings suggest that unique subsets of the immune system can be modeled through iPSC technology for the effective treatment of patients with advanced cancer [1]. Although a lot of studies have been done related to the efficacy and generation of more potent NK cells, very few studies pointed to the role of NK cells in hematopoietic stem cell transplantation (HSCT). A recent study suggested that NK cells negatively regulate the number and function of transplanted HSCs in both humans and mice, and it works in a dose-dependent manner which is mediated by IFN-*γ*. In the same study, it was demonstrated that this negative effect of NK cells was restored by depletion of NK cells or by blocking the IFN-*γ* signaling [2, 25]. Zhu et al. reported that deletion of cytokine-inducible SH2-containing protein (CIS; encoded by the gene CISH) in human iPSC-derived NK cells promoted the expansion of NK cells and increased the cytotoxic activity against multiple tumor cell lines when maintained at low concentrations of cytokine interleukin-15 (IL-15) (Figure 2) [26]. Higher concentrations of IL-15 itself are known to cause toxicities [27, 28]. CIS, belonging to the suppressor of cytokine signaling (SOCS) protein family (other members: SOCS 1–7) [29], negatively regulates IL-15 signaling in NK cells and acts as a checkpoint to regulate NK cell-mediated antitumor immunity in mice [30]. IL-15 is well known to stimulate several functions of the NK cells like differentiation, proliferation, activation, and survival [26, 31–33] and cell metabolism [34–36]. Deletion of CISH in human iPSC-NK cells has been reported to improve metabolic fitness, especially glucose-driven glycolysis and oxidative metabolism which is required for NK cell antitumor and antiviral effector functions [37–40]. However, a study showed that transplantation of mesenchymal stem cells (MSCs) inhibited NK cell proliferation, as well as their cytotoxicity and cytokine production [41].

Signal regulatory protein *α* (SIRP*α*), a regulatory membrane glycoprotein belonging to the SIRP family, acts as an inhibitory receptor. It is mainly expressed by myeloid, neurons, and stem cells, and interacts with a transmembrane protein CD47 which is also known to send the “do not eat me” signal. SIRP*α* and CD47 interactions negatively regulate the effector function of innate immune cells such as host cell phagocytosis. The available body of literature suggests that NK cells do not have SIRP*α* checkpoint in normal healthy individuals. However, Deuse et al. reported that cells from patients had a higher expression of SIRP*α* on NK cells. SIRP*α*, upregulated by IL-2 stimulation, interacts with target cell CD47 in a threshold-dependent manner and counters other stimulatory signals by a number of molecules including IL-2, CD16, or NKG2D. Deletion of SIRP*α* or antibody-mediated blockade augmented the killing capacity of NK cells (Figure 2). They also observed SIRP*α*–CD47 was highly species specific because the overexpression of rhesus monkey CD47 in human MHC-deficient cells prevented cytotoxicity by rhesus NK cells in a xenogeneic setting. This result suggested that elevated expression of CD47 may prevent NK cell–mediated killings cell death in allogeneic and xenogeneic tissues [42].

## 3. ILC1 and Stem Cells

**Figure 3:**
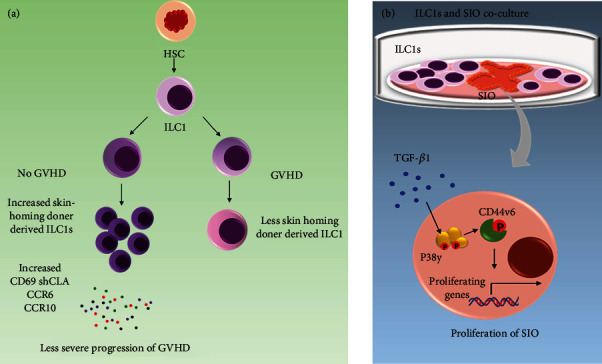
ILC1s in graft versus host disease (GvHD) and in small intestine organoids (SIO): (a) Increase in the expression profiles of ILC1 derived homing markers is an indication of no GvHD whereas their low expression is a marker of progression of severe GvHD. (b) In the coculture conditions, the ILC1s drive expansion of the intestinal epithelial stem cells through TGFb1-p38*γ*-induced phosphorylation, induce CD44v6 expression, and thereby, SIO proliferation.

Both ILC1 and NK cells originate from the same cell lineage, and later, they diverge early in their developmental course. These cells can be differentiated on the basis of the expressed transcription factors, their cytotoxicities, and by the expression of their resident markers. Unlike NK cells, which are cytotoxic, circulating in the bloodstream, killing virus-infected cells, and tumor cells, ILC1s are noncytotoxic or weakly cytotoxic tissue resident cells which function in the defense against infections like viruses and certain bacteria. Being tissue resident, these cells play a major role in graft versus host disease (GVHD) also. The role of ILC1s in GVHD after hematopoietic stem cell transplantation (HSCT) was first time reported by Munneke et al., in 2014 [43]. They monitored the reconstitution of the ILC population after HSCT in 51 acute myeloid leukemia (AML) patients. Their findings suggest that patients who did not develop GVHD had an increased level of skin-homing of donor-derived ILC1s after the HSCs transplantation in PB in comparison to those who developed GVHD. It was further associated with increased expression of activation marker CD69, skin homing CLA (cutaneous leucocyte-associated antigen), and chemokine receptors CCR6 and CCR10 which correlated with less severe progression of GVHD (Figure 3(a)) [43]. However, the functional role of ILC1s could not be ascertained in this study. In 2021. Piperoglou et al. studied the ILC population reconstitution after HSCT in adult and young patients [44]. They found that ILC1 levels in adult patients before the HSCT were the same as that in the healthy controls [44]. However, it increased after the transplant and took one year to come back to the normal range in recipients with the non-GVHD group, whereas in the children it took only 6 months. They have also found a similar association with the homing markers as above mentioned [43, 44]. Both studies suggested that ILC1 population reconstitution was not affected by graft origin or by the amount of CD34 stem cells in the graft. The decrease in the level of circulating ILC1 was associated with the occurrence of GVHD, so that monitoring of ILC1 before HSCT and after reconstitution might be a useful prognostic marker for assessing the patient's risk of developing GVHD. ILC1 accumulation in IBD patients has been reported by Bernink et al. [45]. Jowett et al. studied the distribution of ILC1 in intestinal epithelium in inflammatory bowel disease (IBD) patients. For determining the role of ILC1s, they developed a reductionist coculture system with murine small intestine organoids (SIO). Findings from these coculture conditions revealed that ILC1s drive expansion of the intestinal epithelial stem cells through p38*γ* phosphorylation, which onwards induced CD44v6 expression and SIO proliferation. p38y phosphorylation was induced by TGF-1 secreted by ILC1s (Figure 3(b)) [46]. This finding suggests that ILC1s play a role in intestinal remodeling, which could exacerbate IBD-associated comorbidities when enriched in inflamed intestines. Recently, Bai et al. established the adult mouse liver like fetal liver contained Lin- Sca-1^+^Mac-1^+^ (LSM) cells, which were in significantly higher frequencies than in adult bone marrow (BM), PB, and small intestine lamina propria (siLP). They showed that adult liver containing Lin–Sca-1^+^Mac-1^+^ hematopoietic stem cell (LSM HSC) population was capable to differentiate into tissue-resident liver ILC1s. The study also indicated that IFN-*γ* produced by mature ILC1s also promoted the expansion and differentiation of LSM HSCs into ILC1s but not into NK cells [47]. Further studies are needed to explain why IFN-*γ* derived from these ILC1s support LSM HSC differentiation into ILC1s but not into NK cells. This finding reveals the involvement of extramedullary hematopoiesis to a distinctive regional immune feature within the liver. Though it is not directly related to transplantation, but it is important to know that other groups have been identified umbilical cord blood-derived ILC1-like cells constitute a novel precursor for mature KIR+ NKG2A- NK cells [48]. NK cells derived from ILC1-like cells exhibited key NK cell effector functions including mobilization of cytotoxic granules, the killing of the HLA-deficient target cells, and CD16-mediated ADCC. This finding indicates that this novel ILC1 might be a useful target for NK cell-based stem cell therapy.

## 4. ILC2 and Stem Cells

ILC2s are tissue resident cells involved in the innate response to parasites, such as helminths, and help repair tissue damages. These cells, abundant in tissues like the skin, lung, liver, and gut, are characterized by the production of amphiregulin, and type 2 cytokines including IL-4, IL-5, and IL-13 in response to IL-25, TSLP, and IL-33. Because of their specific cytokine signature, they are considered the innate counterparts of Th2 cells. These cells express characteristic surface markers and receptors for chemokines, which are involved in the distribution of lymphoid cells to specific organ sites. The humans ILC2s expresses CRTH2, ICOS, KLRG1, ST2, CD161, CD127, and CD25 and requires IL-7 for their development [49] and activation of the fundamental transcription factors ROR*α* and GATA3 for the maintenance of their functions. On the other hand, GATA3 deprivation inhibits the development and function of these cells. ILC2s are further classified into subpopulations named natural ILC2s (nILC2s) and inflammatory ILC2s (iILC2s), according to their patterns of response to IL-33 and IL-25 [50]. nILC2s respond to IL-33 in tissues in a natural immune state, while iILC2s respond to IL-25 or to the helminth parasites. Further, nILC2s express more Thy1 and ST2 and have reduced levels of KLRG1 [51]. On the other hand, iILC2s express more KLRG1 and have reduced levels of Thy1 and ST2. In addition, another subpopulation of ILC2s, named as the ILC2^10^ cells, is characterized by their ability to produce IL-10 [52]. ILC2s are also characterized as memory ILC2s which generated a memory response after re-exposure to the allergens and produced type 2 cytokines as well as inflammation [15].

Allogeneic stem cell transplant (allo-SCT) has been used to provide curative therapies for patients with lymphoid malignancies (LM), high-risk acute leukemia, and other malignant diseases [53–55]. Despite the improvements in HLA typing and stem cell donor choices, GVHD remains the major complication of allo-SCT in 30% to 80% of transplanted recipients [56, 57]. Both preclinical transplant models and clinical transplant studies have focused on role for T cells in the pathophysiology of GVHD. Studies on the role of ILCs demonstrated that reduced numbers of circulating CD69^+^ ILC2s were associated with the increased risk of a GVHD in AML patients (22). Bruce et al. studied behavior and role of the ILC2s in the GI track. Their findings demonstrated that when ILC2s were distributed in the GI tract but not in the lung the patients were highly sensitive to conditioning therapies (both chemotherapy and radiation) prior to allo-SCT. More importantly, the study demonstrated that there was a quite limited repopulation of ILC2s from the donor bone marrow in the GI tract. They examined the effects of chemotherapy on ILC2s and found that these were significantly reduced within 24 hours of cyclophosphamide treatment in the LP and MLN. Subsequently, they evaluated the reconstitution of donor and recipient ILC2s for 4 weeks after the allo-SCT and recorded a very reduced number of donor and/or recipient ILC2s in the LP of recipient mice who received irradiation and the bone marrow without donor T cells. The decrease in ILC2s in the LP at day 28 was more as compared to day 1. Cotransplantation of activated ILC2s reduces GVHD and increases recipients' survival due to the production of Th2-cytokine IL13 which suppresses the production of donor T cell proinflammatory cytokines IFN-*γ* and IL17A. These studies strongly suggest that expanded ILC2s may be a potent cellular therapy for the treatment of the lower GI tract GVHD [58]. Recently, it has been reported that third-party ILC2s prevent and treat gastrointestinal (GI) tract GvHD [3, 59]. Previously, it has been reported that an elevated level of soluble ST2 (IL33 receptor) in patients was associated with insufficiency of therapeutic response for GVHD on day 28 and increased mortality after 6 months following the allogeneic stem cell transplantation [60, 61]. The increased levels of ST2 were due to the loss of ILC2s in the GI tract, and it would respond to IL-33 caused inflammations. Cotransplantation of ILC2 decreases the ST2 level in the GI tract as well as the accumulation of IFN-*γ*– and IL-17–producing T cells and diminishes proinflammatory environment [58]. Another study reported that ILC2s promote the self-renewal of intestinal stem cells through IL-13 secretion which initiated the expression of Foxp1 and activated the *β*-catenin pathway [62]. This finding suggests that ILC2s, analogous to ILC3s, may also contribute to epithelial regeneration in the gut and GvHD prevention.

Chemotherapy-induced cell cycle distress cusses' activation of hematopoietic stem and progenitor cells (HPSCs) and promotes bone marrow (BM) regeneration. The role of ILC2 in the recovery of HSPCs from 5-fluorouracil– (5-FU–) induced stress was unknown. However, now we know that this is due to the secretion of granulocyte-macrophage colony-stimulating factor (GM-CSF) by ILC2s. GM-CSF knockout mice treated with 5-FU failed to recover the population of HSPCs which causes severe loss of myeloid lineage cells and lethality, these mice were rescued by transferring BM ILC2s from wild-type mice. This finding might be useful for patients who have recovery problems related to HSPCs during chemotherapy [63].

Stem cell factor (SCF), a cytokine which binds to the receptor tyrosine kinase (c-Kit), is expressed on several myeloid and lymphoid cell types including Type 2 innate lymphoid cells (ILC2). SCF has been known to play roles in the survival of HSCs as in vivo it contributes to the self-renewal and maintenance of HSCs [64]. The importance of the SCF/c-Kit interaction in ILC has been further reported by Fonseca et al., and they found significantly increased SCF in the serum of both asthmatic patients as well as in the mouse model of the disease in comparison to the non-asthmatic controls. Further analysis revealed that the expression of SCF248 isoform was overexpressed in the lungs of allergic mice, whereas the expression level of the SCF220 isoform was unaltered when compared to naive mice [65]. Therefore, they concluded that the higher circulating levels of SCF in the serum were due to the increased expression of SCF248 in the lungs. Mice treated with SCF248 monoclonal antibody had mitigated the development of chronic asthmatic symptoms by decreasing the number of mast cells, ILC2s, and eosinophils, as well as by reducing the associated pathogenic cytokine responses [65]. To know the effects of SCF on the ILC2s, they stimulated the sorted ILC2 cells with recombinant murine SCF (rSCF). They observed that the cells stimulated with SCF had significantly increased expression of inhibitor of DNA binding 2 (ID2) and GATA3 [65]. Both transcription factors are important for the maintenance and differentiation of the ILC2 cells. This data suggested that the SCF is contributing to the development and maintenance of ILC2 phenotypes. Next, they studied the activation role of SCF on ILC2 effector functions and found that rSCF-stimulated sorted cells had increased levels of type 2 cytokines as well as of ILC2 expressed receptors IL9R and IL17RB (IL25R) [65].

**Figure 4:**
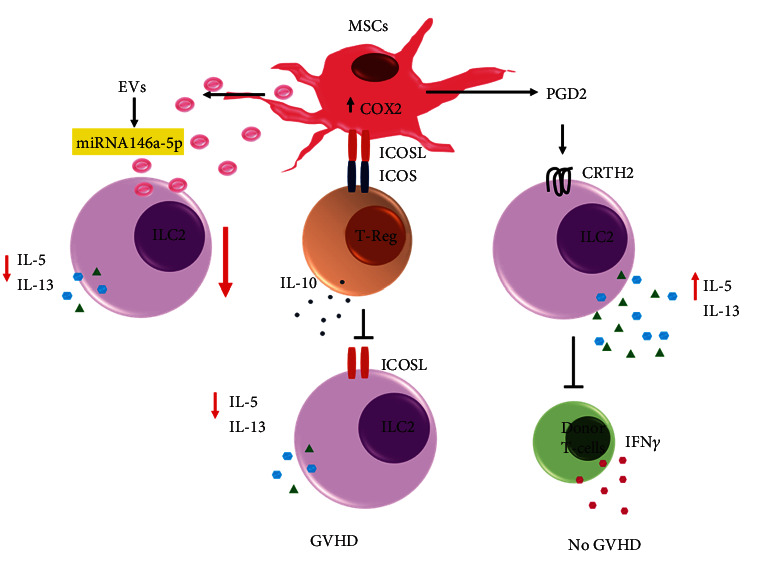
ILC2s and mesenchymal stem cells (MSCs) in GvHD and inflammation: MSCs express cyclooxygenase and prostaglandin D2 (PGD2), a ligand for prostaglandin D2 receptor 2, which binds to ILC2 and induces production of type 2 cytokines and inhibits IFN-*γ* production. MSCs also bind with Treg and induce the expression of IL10 which inhibits ILC2 and progression of GvHD. MSC-derived small extracellular vesicles (sEV) express miR146a-5p which inhibits ILC2 and production of type 2 cytokines.

Recent publications show that stem cells play an important role in the regulation of ILC2s. Findings on ILC2s isolated from both healthy or allergic rhinitis patients cocultured with pluripotent stem cell-derived MSCs (iPSC-MSCs) in the presence of IL25+IL33 showed decreased levels of type 2 cytokines IL5+, IL13+, and IL9+ demonstrate that stem cells inhibit the effector functions of ILC2s (Figure 4). In a subsequent study, they found that this inhibitory effect of MSCs on ILC2s was mediated by the regulatory T (Treg) cells which were regulated through ICOS-ICOSL interactions (Figure 4) [66]. Previously, it has been reported that MSCs from acute myeloid leukemia (AML) patients or from normal individuals overexpressing COX2 had elevated secretion of prostaglandin D2 (PGD2). PGD2 is a ligand for chemoattractant receptor homologous (CRTH2) receptor which is expressed by the ILC2 and Th2 cells. PGD2-CRTH2 interaction is important for the activation and function of ILC2s and production of type 2 cytokines. Type 2 cytokines are important for the inhibition of donor IFN-*γ* which produce by the donor Th1 cells and causes GVHDs (Figure 4). PGD-mediated activation of the ILC2-Treg axis is involved in the proliferation of normal and malignant HSPCs [66]. Recently, it has been reported that neuromesenchymal stem cells regulate ILC2 function and activation in obesity via a brain–adipose circuit [4, 67].

Unusual or abnormal actions of ILC2s have been recorded in certain pathological conditions. Stem cells or derived small extracellular vesicles are being tried to counteract certain ILC2-mediated pathological conditions. The effectiveness of small extracellular vesicles (sEV) derived from iPSC-MSCs on patients with allergic rhinitis and in mouse ILC2-dominant asthma model was reported by Fang et al. [68]. They developed a standardized scalable protocol of anion exchange chromatography for isolation of MSC-sEV and identified MSC-sEV by flow cytometry which expressed surface markers CD9/CD63/CD81 but did not express the general markers of MSCs like CD44, CD146, CD73, CD90, and CD105 [68]. To evaluate the effects of MSC-sEV on the functions of ILC2s, they isolated human PBMCs from patients with allergic rhinitis, cultured, and then activated them with IL-2/25/33 in the presence or absence of MSC-sEV. Cultured of PBMCs or sorted ILCs in presence of MSC-sEV had reduced expression of IL9+ and IL13+ ILC2 as compared to the control. Administration of MSC-sEV in a mouse model of asthma showed reduced levels of ILC2, type 2 cytokine expression, inflammatory cell infiltration, and mucus production in the lung and had alleviation in the airway hyperresponsiveness [68]. RNA sequencing analysis revealed that MSC-sEV had highly enriched miR-146a-5p, known to inhibit the function of activated ILC2 [69, 70]. So, they concluded that the inhibition of ILC2s, type 2 cytokine production, and airway inflammation in presence of MSc-sEV was mediated by miR-146a-5p (Figure 4). The findings suggest that MSC-sEV could be a novel cell-free strategy for the treatment of allergic, inflammatory, and asthmatic disease. Recently two subsets of ILC2s were identified in islet allografts of IL-33-treated mice: IL-10 producing ILC2s (ILC2^10^) and non-IL-10 producing ILC2s (non-ILC^10^). Intravenous transfer of ILC2^10^ cells, but not non-ILC^10^, prolonged islet allograft survival in an IL-10-dependent manner. Locally transferred ILC2^10^ cells led to long-term islet graft survival, suggesting that ILC2^10^ cells are required within the allograft for maximal suppressive effect and graft protection. This study has uncovered a major protective role of ILC2^10^ in islet transplantation which could have the potential to be used as a therapeutic strategy [71].

## 5. ILC3 and Stem Cells

ILC3s constitute a group of cells imparting innate immunity which participates in defense mechanisms to mucous membranes or mucosa and represents a defense mechanism against extracellular parasites, bacteria, and fungi. These cells are involved in the maintenance of intestinal microorganisms-host homeostasis and in the regulation of host-commensal mutualism. ILC3s generally are characterized by the presence of surface markers Lin- CD127+ CD3-, transcription factor RoRyt, signature cytokines IL-17 (Th17) and IL22 (Th22 cells), and chemokine receptor CCR6. Initially, ILC3s were divided into two subsets: lymphoid tissue inducer cells- (LTi-) like ILC3s and natural cytotoxicity receptor (NCR) ILC3s (NKp46 in mice and NKp44 in humans) which are developmentally, phenotypically, and functionally different [4, 5, 16]. The sets of surface markers in different sets of ILC3s are species-specific. Lti-like ILC3 express LIN-, CD1a-, CD11c-, CD34-, CD123-, BDCA2-, Fc*ε*RI-, TCR*αβ*-, TCR*γδ*-, IL-7R*α*hi, CD45int, ROR*γ*t (also CD4-, CD94-, CD7, nd CD161) in humans, and produce signature cytokines LT*α*, LT*β*, IL-17A, IL-22 [4, 13, 72]. NCR+ ILC3 cells are further divided into two subtypes, i.e., NKp46+ ILC3 and NKp44. NCR+ ILC3 express LIN-, CD1a-, CD11c-, CD34-, CD123-, BDCA2-, Fc*ε*RI-, TCR*αβ*-, TCR*γδ*-, CD56, IL-7R*α*, NKp30, NKp44, NKp46, AHR, and ROR*γ*t and produce cytokine IL22 [4, 13, 72]. On the other hand, NCR-ILC3 express CCR6, CD4-, CD16-, CD94-,c-kit, IL-7R*α*, NKG2D-, NKp44lo/-, NKp46-, RANKL, AHR, and ROR*γ*t and cytokines IFN-*γ*, IL-17A, IL-22, and TNF-*α* [4, 13, 72]. The ability of ILC3 cells to promote tissue repair, maintain the tissue integrity, and defend against pathogens could be useful in patients receiving HSCT or those having severe GVHD. It has been reported that a transient increase in the levels of circulating NCR^+^ ILC3 correlates well with reduced incidences of GvHD [43]. It has been shown that CD34^+^ cells, used as a source of hematopoietic precursors in HSCT, derived from different sources including BM, PB of G-CSF-mobilized in donors or from umbilical cord blood (UCB), generate NK and ILC3 cells in different proportions. ILC3s are more represented in the lymphoid progenies of CD34^+^ precursors derived from UCB or BM. In addition, a negative effect on the ILC3 generation is exerted by G-CSF, as shown by *in vitro* studies [73]. IL-1*β* has been shown to affect the differentiation of CD34^+^ precursors towards ILC3, favoring NK cell development, suggesting that inflammatory responses may interfere with ILC3 generation [74, 75].

ILC3s regulate intestinal stem cell maintenance and subsequently help tissue repair in cases of acute insults. ILC3-produced IL-22 was considered to be important for stem cell protection [76]. However, it was recently reported that ILC3-driven epithelial proliferation and tissue regeneration are independent of IL-22. It has been demonstrated that ILC3s amplify the magnitude of Hippo-Yap1 signaling in intestinal crypt cells, ensuring adequate initiation of tissue repair, and preventing excessive pathology. These findings reveal that ILC3-driven intestinal repair entails distinct transcriptional networks to control stem cell maintenance and epithelial regeneration, which implies that tissue repair and crypt proliferation can be influenced by targeting innate immune cells in addition to the well-established effects of IL-22. The ILC3-driven tissue repair is Stat3 independent which involves activation of Src family kinases [77].

Kang et al. in 2020 reported the protective role of type 3 NKp44^+^ILCs (ILC3s) which are significantly diminished in newly transplanted allografts in comparison to allografts after 6 months, where pro-inflammatory type 1 NKp44^−^ILCs (ILC1s) were higher in intestinal transplants [78]. Moreover, serial immune monitoring revealed that in healthy allografts, protective ILC3s repopulate between 2 and 4 weeks postoperatively, but in allografts being rejected they remain diminished. NKp44^+^ILC3 cells produce protective interleukin-22 (IL-22), whereas ILC1s produce proinflammatory interferon-gamma (IFN-*γ*) and tumor necrosis factor-alpha (TNF-*α*). Intestinal grafts carry a large donor lymphoid load that is replaced by the cells of the recipient. The dynamics of this process may influence the tolerance, rejection, or graft-versus-host disease. Gómez-Massa et al. analyzed the distribution and turnover of T and B (Lin+) lymphocytes, natural killer (NK), and helper innate lymphoid cells (hILC) in the intestinal epithelium (IEp) and lamina propia (LP) from a long-term cohort of eight intestinal recipients and from a single patient monitored closely during the first 8 months posttransplant (posTx) [79]. Long-term intestinal grafts showed significantly higher %hILC than native bowels in IEp and LP until 10 years posTx and recovery to normal levels was observed afterward. They also observed an imbalance between hILC subsets in IEp (increase in ILC1s and decrease in ILC3s) that persisted along posTx time even when %hILC was like native bowels. Regarding hILC origin, they detected the presence of donor cells even 13 years posTx. However, this chimerism was significantly lower than in Lin+ and NK populations. The findings based on the observations from the patients monitored in the early posTx period showed that recipient hILC repopulate earlier and faster than Lin+ cells. The increases in ILC1s are often related to rejection and infection episodes [79]. This finding shows that ILCs might play a key role in the regulation of intestinal transplant graft homeostasis and could serve as sentinels for early recognition of allograft rejection and, therefore, could be a target for future therapies.

## 6. The Generation of NK Cells and ILCs from CD34 + HSC

The most intriguing aspect of cancer therapy has been the resistance developed in the cancer cells to the drugs. Recent advances in the field of immunotherapy, in which NK cells play a very important role, have offered hope to cancer patients. This innovative approach is based on the idea of harnessing specific cells of the immune system to target tumor cells. The NK cell-based immunotherapy has emerged as a promising therapeutic approach against solid tumors and hematological malignancies. These cells are innate lymphocytes with an array of functional competencies, including anticancer, antiviral, and antigraft-vs.-host disease potentials. For this purpose, the NK cells are isolated and propagated. To achieve this, CD34^+^ cells isolated from BM, PB, or UCB are cultured with SCF, FMS-like tyrosine kinase ligand (Flt3-L), IL-7, and IL-15 Cytokine-Mix (hereinafter, referred to as c-Mix), and analyzed by flow cytometry (FC) at different time intervals [80]. It has been noticed that the differentiation pattern of the cell population varies according to the source tissues they are isolated from. Generally, in these cells, CD34 expression progressively decreases during the in vitro culture. Oberoi et al. reported a refined approach based on ex vivo culture of PB-CD34^+^ cells with optimized cytokine cocktails that reliably generate functionally mature NK cells, as assessed by analyzing NK-cell-associated surface markers and by cytotoxic activity. To further enhance NK cell expansion, they generated K562 feeder cells coexpressing 4-1BB ligand and membrane-anchored IL-15 and IL-21. Coculture of PB-derived NK cells and ex vivo differentiated from HSCs NK cells with these feeder cells had dramatically improved NK cell expansion [81]. This finding suggested mobilized PB-CD34^+^ cells expanded and differentiated according to this two-step protocol as a promising source for the generation of allogeneic NK cells for adoptive cancer immunotherapy.

In stem cells of BM origin, CD19^+^ cells are present while no CD3^+^ cells are detected. At day (d) 0, PB HSCs have CD33^+^, CD13^+^, and CD115^+^, suggesting their commitment towards the myeloid lineage. However, after 10 days, cells from all sources in cultures contain CD33^+^CD14^+^ cells which become predominant by d 30. Around day 10, lineage (lin)^−^CD56^+^CD161^+^ ILCs are detectable only in BM cultures which increase up to 50% of cells by day 30, while they appear in PB and UCB cultures only at later time points. Notably, although the percentages of ILCs are significantly higher in BM cultures, BM HSCs display the lowest expansion rate. Thus, when considering the absolute numbers of CD56^+^CD161^+^ ILCs, UCB cultures give the highest recovery. Further, analysis of expressed transcription factors on CD56 and CD161 antigens expressing cells, the antigens expressed by both NK cells and ILC3s, demonstrate that the majority of CD56^+^CD161^+^ cells expressed receptor activator of nuclear factor kappa-B ligand (RANKL) and ROR*γ*t, thus representing ILC3s [82].

Upon stimulation, the CD56^+^CD161^+^ cells mainly produce ILC3s while the percentage of IFN-*γ*-producing cells, i.e., NK cells, remains low. Thus, CD34^+^ cells from BM and UCB HSCs display a better capability of in vitro differentiation towards ILCs than those from PB. It is conceivable that the preferential myeloid commitment of PB CD34^+^ cells may be a consequence of G-CSF-induced mobilization [73]. NK cells, also a component of the innate immunity system, are innate lymphocytes characterized by the expression of nuclear factor interleukin 3 regulated (NFIL3 or E4BP4), eomesodermin (EOMES) transcription factors (TFs), and by the ability to exert cytolytic activity, and also by the ability to release IFN-*γ*. In the haploidentical hematopoietic stem cell transplantation (haplo-HSCT) settings, donor-derived CD34+ NK cells play a major role in the control of leukemic relapses. Therefore, it is of utmost importance to define the cytokines that influence NK cell differentiation from CD34+ precursors. A recent study on the analysis of the effects of IL-1*β* on NK-cell differentiation of umbilical cord blood (UCB) CD34+ cells revealed that while IL-1*β* inhibited CD161+CD56+ cell proliferation, it increased expression of LFA-1, CD94/NKG2A, KIRs, and perforin in CD56+ cells. In addition, within the CD161+CD56+IL-1RI+LFA-1 cell fraction (representing group 3 innate lymphoid cells, ILC3-like cells), a significant increase in the levels of EOMES, NKp46, and CD94/NKG2A receptors, cytolytic granules, and IFN-*γ* was detected. This increase paralleled the decreases in the levels of related orphan receptors (ROR*γ*t) TF, NKp44 expression, and IL-22 production. The data suggest that IL-1*β* inhibits ILC3s while favoring NK cell maturation. Since in haplo-HSCT conditioning regimen, infections, or residual leukemia cells may induce IL-1*β* production, this may influence the NK/ILC3 development from donor-derived CD34+ cells [74]. Shokouhifar et al. found that the differentiation of ex vivo expanded CD34+ cells through manipulation of RAS/MAPK, IGF-1R, and TGF-*β* signaling pathways is an efficient approach for generating functional NK cells that can be used for cancer immunotherapy [83]. CD34^+^-HSPC cultured in the absence or in the presence of the EZH1/2 inhibitor UNC1999 and EZH2 inhibitor GSK126 showed that UNC1999 and GSK126 increased CD56^+^ cell proliferation in comparison to the control. However, UNC1999 and GSK 126 favored the proliferation of no-cytotoxic CD56^+^ILC3, evident by the early expression of the aryl hydrocarbon receptor (AHR) and ROR-*γ*t transcription factors [84]. These results indicate towards novel epigenetic mechanisms involved in the modulation of NK cell maturation that may provide new tools for designing NK cell-based immunotherapy [84].

Human cytomegalovirus (HCMV) is highly prevalent in most populations worldwide which has a major influence on shaping the human immune system. In a recent report, the system was modified by infecting MSCs with HCMV to study the effect of virus infection on NK/ILC development. The report demonstrated that cord blood-derived hematopoietic progenitor cells were successfully differentiated into mature CD56+CD94+NKG2A+ NK cells from HCMV-infected MSCs having significantly higher antiviral cytokine production ability in comparison to NK cells developing from noninfected MSCs. Furthermore, the generation of ILC3s, characterized by expression of the signature transcription factor RAR-related orphan receptor gamma (ROR*γ*t) and by the production of IL-22, was strongly impaired by HCMV infection. These observations are of significant clinical relevance given that ILC3s are associated with protection from graft-versus-host disease (GvHD) following stem cell transplantation. Also, the finding demonstrates that HCMV-mediated reactivation could be associated with increased incidences of GvHD [85]. A recent report demonstrates that the differentiation of hematopoietic stem cells towards NK cells and away from common ILC precursors can be achieved by glucocorticoids, a finding that can be used for favoring the production of NK cells [86].

## 7. Conclusion

The stem cell therapy industry is growing at a very fast pace because it is playing a very important role in offering hope to a section of patients who have lost hope from existing medical knowledge. In past decades, several studies have been carried out with T and NK cell-based immunotherapy, but very little information is available regarding the ILC1, ILC2, and ILC3 as this is a very new field. These cells are known for maintaining homeostasis, but no mechanistic study is available regarding their role during stem cell therapy. Production and targeting the NK cells using stem cells have very high potentials to be used in the field of cancer immunotherapy.

## Data Availability

Not applicable since it is a review article.
